# Comparative Study Between Conventional Landmark Versus Ultrasound-Guided Paravertebral Block in Patients Undergoing Laparoscopic Cholecystectomy: A Randomized Controlled Study

**DOI:** 10.7759/cureus.36768

**Published:** 2023-03-27

**Authors:** Manoj Kumar, Jay Brijesh Singh Yadav, Amit K Singh, Amit Kumar, Dheer Singh

**Affiliations:** 1 Anaesthesiology, Uttar Pradesh University of Medical Sciences, Etawah, IND

**Keywords:** postoperative analgesia, laparoscopic cholecystectomy, ultrasound guided, conventional landmark, bupivacaine, paravertebral block

## Abstract

Introduction: Thoracic paravertebral block (TPVB) has emerged as an effective and safe regional technique for providing postoperative analgesia. We aimed to compare the ease and efficacy of conventional landmark and ultrasound-guided (USG) paravertebral blocks for postoperative analgesia in patients undergoing laparoscopic cholecystectomy.

Methods: This was a randomized controlled study. Seventy-six patients of either sex, age 18-40 years, body mass index (BMI) 18-29 kg/m^2^, American Society of Anesthesiologists physical status classifications I and II posted for elective laparoscopic cholecystectomy under general anesthesia were randomly allocated into two groups of 38 each. Patients in group A were administered a paravertebral block using the anatomical landmark technique (ALT), and group B using an ultrasound-guided paravertebral block in the sitting position. In both groups, 20 ml of 0.5% bupivacaine injection was administered at the T7 vertebral level on the right side. The primary outcome was the first-pass success rate. Secondary outcomes were the number of passes and attempts, duration of analgesia, visual analog scale (VAS) score for pain during 24 h postoperatively and complications if any, were recorded.

Results: No patients were excluded in the study. Demographic characteristics were comparable in both groups. The number of passes was less in group B (1.45±0.5) compared to group A (2.42±0.95) and was reported to be statistically significant (p = 0.001). The number of attempts was less in group B (1.00±0) as compared to group A (1.29±0.46) and was statistically significant (p = 0.001). The duration of analgesia was longer in group B (530.00±326.33 minutes) compared to group A (345.60±252.95 minutes) and was observed to be statistically significant (p<0.05). The VAS score was significantly lower in group B (1.87±0.78, 2.24 ±0.82) compared to group A (2.42±0.72, 3.13±1.07) at the second^ ^and fourth hours, respectively (p = 0.001).

Conclusion: We concluded that paravertebral block using an ultrasound-guided technique is more efficacious than the conventional landmark technique for postoperative analgesia in patients undergoing laparoscopic cholecystectomy.

## Introduction

Laparoscopic cholecystectomy is nowadays a widely accepted surgery for cholelithiasis. Therefore, the needs and comfort of patients are equally important as compared to maintaining the quality of the surgery and its outcome. The majority of the patients suffer from severe abdominal and shoulder pain in the postoperative period and require strong analgesia after laparoscopic surgery [1-3]. The main cause of postoperative pain in laparoscopic cholecystectomy occurs due to pneumoperitoneum, cholecystectomy, and incision sites [4]. Pain is an important factor affecting morbidity related to the cardiovascular system, pulmonary system, and emotional state in surgical patients during the postoperative period. Appropriate and effective treatment of pain may reduce the incidence of complications, recovery time, hospital stay, and health costs [5].

Systemic opioids, nonsteroidal anti-inflammatory drugs (NSAIDs), intraperitoneal local anesthetics (LA), epidural blocks, and paravertebral blocks (PVBs) are commonly employed for the treatment of postoperative pain. The use of systemic opioids is associated with side effects, such as nausea, vomiting, constipation, and respiratory depression [6,7]. On the other hand, nonsteroidal anti-inflammatory drugs may cause gastrointestinal problems. Epidural analgesia is effective in relieving pain and promoting the recovery of pulmonary function. However, it carries the risk of dural puncture, nerve lesions, bleeding, infection, hypotension, bradycardia, as well as urinary retention.

Thoracic paravertebral block (TPVB), first described by Hugo Sellheim in 1905, is the administration of local anesthetic into the wedge-shaped space on the anterolateral thoracic spine to provide muscle relaxation and thoracoabdominal analgesia. Thoracic paravertebral block (TPVB) provides better analgesia, decreases analgesic requirement and postoperative nausea and vomiting, less complications, and improved quality of recovery in patients undergoing many types of operation, including thoracotomy, abdominal surgery, breast surgery, pulmonary surgery, and herniorrhaphy and patients with thoracoabdominal trauma [8,9]. Paravertebral block can be performed with two types of technique, the older one anatomical landmark-based and the newer one ultrasound-guided (USG) technique. In the anatomical landmark technique (ALT), the transverse process (TP) is an important landmark in the thoracic paravertebral block. However, the transverse process is neither visible nor palpable, its location is unknown until the block needle encounters the bone [10]. Sometimes, it may be difficult for obese patients. In the last decade, many approaches of ultrasound-guided (USG) PVB block have been described that involve visualizing the hyperechoic TP, the underlying hypoechoic paravertebral space, and anterior displacement of the pleura on local anesthetic (LA) administration [11]. Ultrasonographic measurements of the distances from the skin to the transverse process and to the parietal pleura are useful for calculating the required depth of needle insertion. USG PVB may improve the success rate of the block and decrease potential complications [12].

In our literature review, we found limited studies comparing the ultrasound-guided with the landmark technique of PVB for postoperative analgesia in patients undergoing laparoscopic cholecystectomy. Hence, we planned a study to investigate and compare the conventional landmark versus ultrasound-guided paravertebral block for postoperative analgesia in patients undergoing laparoscopic cholecystectomy.

## Materials and methods

This study was conducted after approval from the Institutional Ethical Committee of the University (Ref No. 1881/UPUMS/Dean(M)/Ethical/20-21, EC No. 149/2020-21). Informed & written consent was obtained.

Inclusion and exclusion criteria

Patients with American Society of Anesthesiologists-Physical Status (ASA-PS) classification I and II, age 18 to 40 years, and body mass index (BMI) 18-29 kg/m^2^ scheduled to undergo elective laparoscopic cholecystectomy under general anesthesia were included in the study. Patients suffering from any medical illness like cardio-pulmonary, hepato-renal, or metabolic disorders or patients with mental problems having difficulty understanding the scoring for pain were excluded from the study.

Sample size calculation and randomization

Sample size calculation was done based on a previous study assuming a 5% significance level with a 95% confidence interval and a power of 80% using Statistical Package for Social Sciences (SPSS version 20.0) software (IBM Corporation, Armonk, New York, USA) [13]. The sample size comes out to be 76 patients (38 patients per group).

Sample size calculation equation:

$n=2\times [(Z_{\alpha /2}+Z_{1-\beta })^{2}\times \sigma^{2} ]/(\mu_{1}-\mu_{2} )^{2}$,

n = 2 × [(1.96 + 0.842)^2^ × (3.1)^2^/4,

 = 37.72 round off to 38,

where n is the sample size per group, Z_α/2_ = 1.96, standard normal z-value for significance level α = 0.05, Z_1-__β_ = 0.842, standard normal z-value for the power of 80%, which is 0.80, µ_1_ = 3.3, µ_2_ = 1.3, σ = 3.1, standard deviation.

Randomization was done using the sealed opaque envelope technique, and patients were divided into two groups to receive paravertebral blocks using the conventional landmark technique (group A) and ultrasound-guided technique (group B). The anesthesiologist who administered the block was not involved in data collection. The preanesthetic check-up was done one day before, which included a detailed history, general physical and systemic examination, and required laboratory investigations. Patients enrolled in the study were instructed on how to judge the intensity of pain using a visual analog scale (VAS), a scale of 0-10 (0 = no pain and 10 = worst pain). All patients received an oral tablet of 0.25 mg of alprazolam on the night before surgery. On the day of surgery, patients were transferred to the operation room, where baseline standard parameters were recorded, such as heart rate, noninvasive blood pressure, electrocardiogram, and peripheral saturation of oxygen.

Procedure

Anatomical Landmark Technique

After all standard sterile precautions and LA infiltration (2-4 mL 1% lignocaine), a PVB block was performed at T7 with a 22-gauge spinal needle. After hitting the transverse process (TP), the needle was withdrawn and advanced 1 cm beyond the cephalad edge of the TP. Simultaneously, loss of resistance to air was assessed. In case the needle hit bone, it was redirected along the caudad edge of the TP, and thereafter, LA 20 ml of 0.5% bupivacaine was administered at the T7 level.

Ultrasound-Guided Technique

A 5 to 12 Hz linear array ultrasound probe in the transverse plane was used first to identify each spinous process, then slide laterally to visualize TP. The probe was rotated 90 degrees clockwise to obtain a parasagittal oblique (PSOQ) view of the PVB space, with a delineation of the superior costotransverse ligament. A 22-gauge spinal needle was inserted from the caudad to the cranial direction in the plane. After confirming anterior displacement of the parietal pleura with an injection of 1 to 2 mL of saline, LA 20 ml of 0.5% bupivacaine was administered at the T7 level.

**Figure 1:**
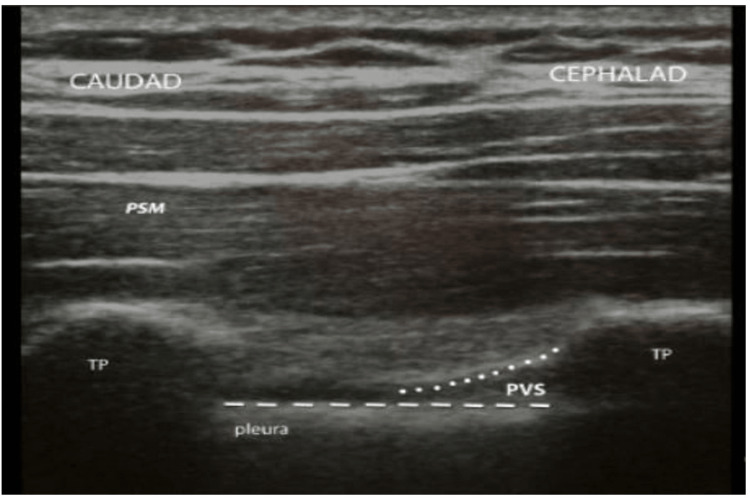
Ultrasound views of the paravertebral space (PVS): sagittal ultrasound view of the PVS. TP: transverse process, PSM: paraspinal muscle.

The number of passes (i.e., the number of forward progressions of the needle with redirection in a given interspinous space but without withdrawal from the skin) and insertion attempts (i.e., the number of times the needle was removed from the skin and reinserted). The first pass success is defined as a needle entering the subarachnoid space in one attempt without changing direction. The number of passes and attempts were recorded until the completion of the procedure. We arbitrarily defined block success as loss of pinprick sensation in three or more ipsilateral vertebral dermatomes. Then, analgesia using the loss of pinprick sensation was checked 15-20 minutes after the institution of the block to elicit the extent of the somatic blockade. Failed PVB was defined as the inability to identify the sensory block in the dermatome between T6 and T8, 20 minutes after the initiation of block (Figure 1).

Standard general anesthesia protocols were followed in both groups. Patients were premedicated with injections of glycopyrrolate (5 mcg kg^−1^), midazolam (0.05 mg kg^−1^), and fentanyl (2 mcg kg^−1^). Preoxygenation was done with 100% oxygen for three minutes. Induction was done with an injection of propofol 2 mg kg^−1^ and intubation was facilitated using an injection of vecuronium (0.1 mg kg^−1^). Anesthesia was maintained with 50% of nitrous oxide in oxygen and isoflurane and was titrated according to the respective group protocol, along with an injection of vecuronium (0.02 mg/kg^−1^) at a maintenance dose as required. Injection paracetamol (15 mg kg^−1^) was given as analgesia during the intraoperative period in both groups.

Study outcomes

The primary outcome in this study was to compare the first-pass success rate of PVB in both groups. Secondary outcomes were the number of passes, number of attempts, procedure time, time to the first analgesic request, duration of surgery, and pain assessment using the VAS scale. Hemodynamic parameters such as heart rate, blood pressure, and peripheral oxygen saturation were recorded preoperatively, after administration of a block (baseline), followed by every 10 minutes intraoperatively and postoperatively (0, 10th, 20th, 30th, 45th, 60th minutes) for the first hour and thereafter at 2, 4, 6, 12, and 24 h. After the completion of surgery, the pain was assessed using the visual analog scale on 0-10 (0 = no pain, 10 = worst possible pain) at 0, 1, 2, 4, 6, 8, 12, and 24 h. Time to first rescue analgesic request was noted. When the VAS score reached >3, postoperative rescue analgesia (diclofenac 1.5 mg/kg) was administered intravenously.

Statistical analysis

Data were analyzed using the Statistical Package for Social Sciences (SPSS) version 20.0 (IBM Corporation, Armonk, New York, USA). Quantitative data were summarized as mean±standard deviation and analyzed using an unpaired t-test. The qualitative data were expressed as numbers and percentages and were analyzed by the Chi-square test. P-value <0.05 was considered statistically significant and highly significant if P value <0.001.

## Results

**Figure 2:**
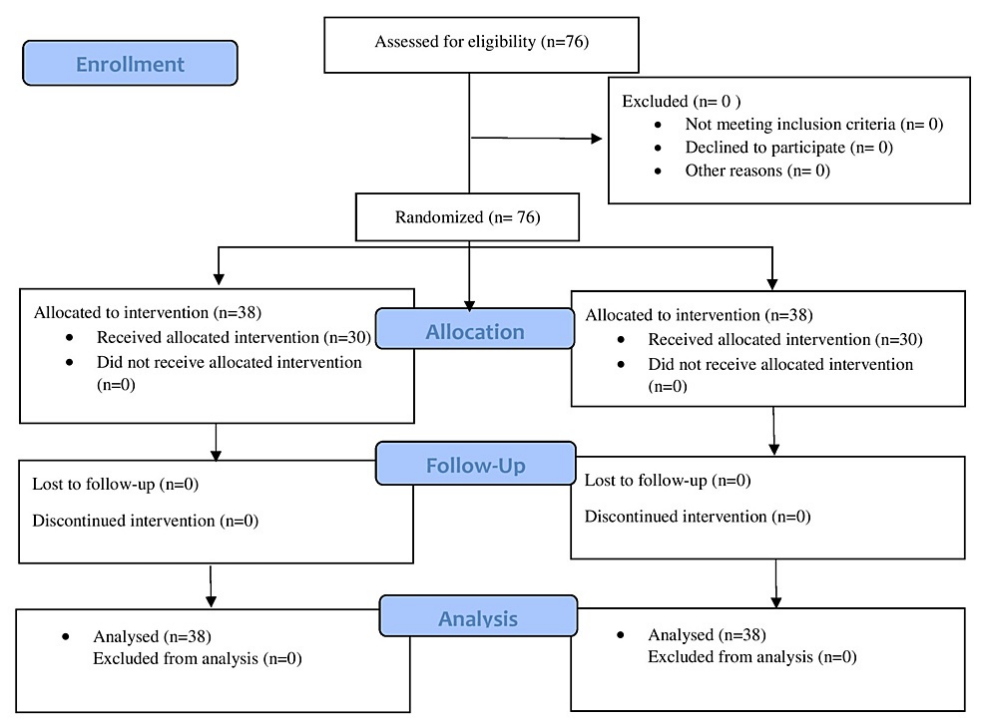
CONSORT flow diagram. CONSORT: Consolidated Standards of Reporting Trials.

No patients were excluded in our study. Seventy-six patients who planned to undergo laparoscopic cholecystectomy were randomly divided into two groups of 38 each (Figure 2).

Both groups were comparable in terms of demographic data and clinical characteristics. Statistically, no significant difference was observed in terms of age, gender, ASA physical status classification, and duration of surgery (Table 1).

**Table 1:** Demographic characteristics. ASA-PS: American Society of Anesthesiologists-Physical Status.

Parameters	Group A (n = 38)	Group B (n = 38)	P-value
Age (years)	28.74±6.44	28.74±5.72	0.500
Sex (male/female)	5/33	5/33	0.500
ASA-PS I/II	35/3	35/3	0.500
Duration of surgery (minutes)	40.53±8.68	36.84±10.68	0.052

**Figure 3:**
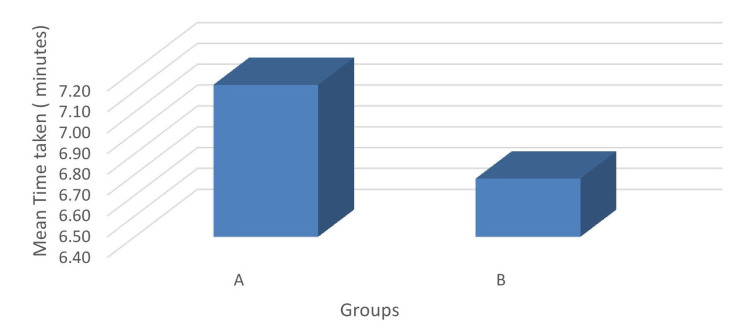
Comparison of the mean time taken to perform the paravertebral block.

The mean time taken for performing the paravertebral block was 7.13±2.16 minutes and 6.68±2.05 minutes in group A and group B, respectively. Both groups were comparable with respect to the time taken to perform the paravertebral block (Figure 3).

The first-pass success rate (55.26% vs. 18.42%) and success rate within two passes (44.74% vs. 34.21%) were higher in group B compared to group A. During the intergroup comparison, the mean values were significantly higher during the first pass success rate (P<0.001) (Table 2). The first attempt success rate was 100% in group B patients compared to 71.05% in group A patients and was highly significant between the groups (P<0.001) (Table 2).

**Table 2:** Comparison of number of passes and number of attempts between the groups.

Number of passes	Group A (n = 38)		Group B (n = 38)		P-value
	n	%	n	%	
1	7	18.42	21	55.26	<0.001
2	13	34.21	17	44.74	0.174
3	13	34.21	0	0.00	<0.001
4	5	13.16	0	0.00	<0.001
Number of attempts	Group A (n = 38)		Group B (n = 38)		P-value
	n	%	n	%	
1	27	71.05	38	100	<0.001
2	11	28.95	0	0.00	

**Figure 4:**
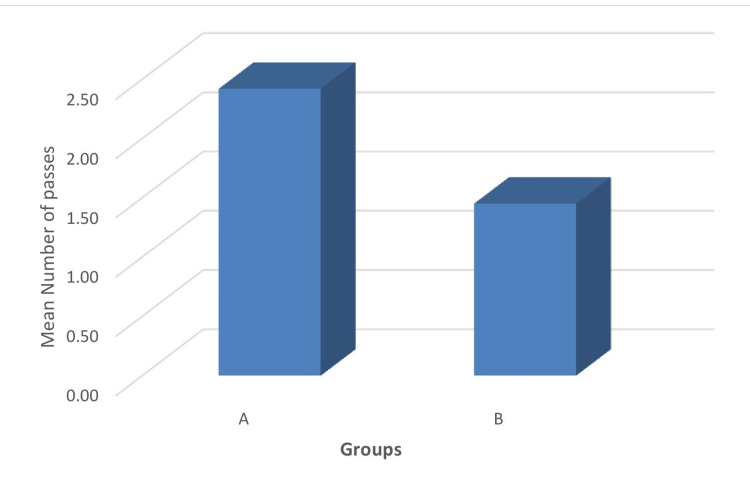
Comparison of the mean number of passes between the groups.

The mean number of passes was less in group B (1.45±0.5) compared to group A (2.42±0.95). The average number of passes in group B was approximately 0.60 times that of group A (Figure 4). During the intergroup comparison, the mean values were found to be statistically significant between the groups (p = 0.001).

**Figure 5:**
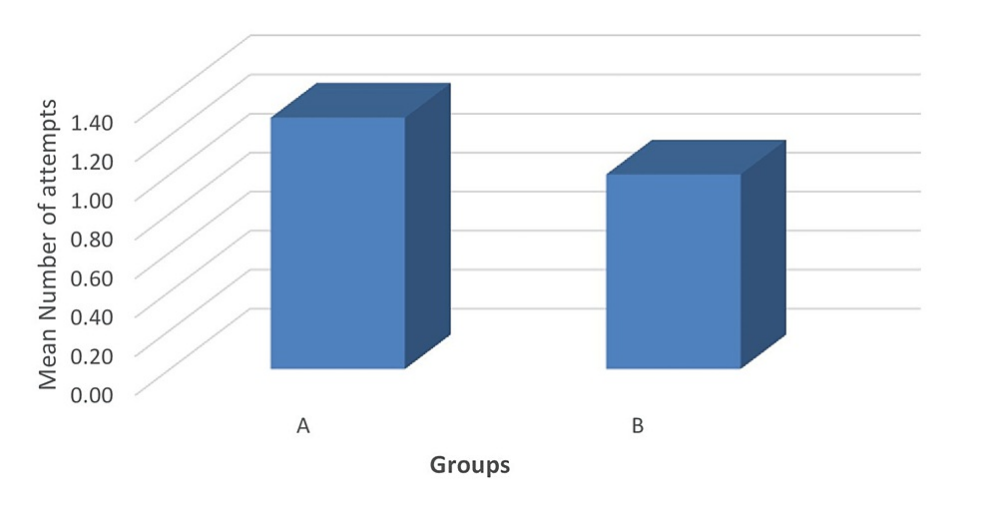
Comparison of the mean number of attempts between the groups.

The mean number of attempts was significantly less in group B (1.00±0) compared to group A (1.29±0.46), (p<0.05). The average number of passes in group B was approximately 0.77 times that of group A (Figure 5). The result was found statistically significant between the groups (p = 0.001).

The mean VAS score was lower in group B than group A at all time intervals. However, at the second and fourth hours, VAS scores were less in group B (1.87±0.78, 2.24±0.82) compared to group A (2.42±0.72, 3.13±1.07), respectively, and were observed to be highly significant (p = 0.001) (Table 3).

**Table 3:** Comparison of mean visual analog score between the groups.

Visual analog score	Group A (n = 38)	Group B (n = 38)	P-value
	Mean±SD	Mean±SD	
0 h	1.00±0.99	0.95±0.96	0.407
1 h	1.61±1.1	1.39±1	0.193
2 h	2.42±0.72	1.87±0.78	0.001
4 h	3.13±1.07	2.24±0.82	0.001
6 h	2.71±0.77	2.47±0.76	0.091
8 h	2.79±0.74	2.58±0.72	0.107
12 h	2.45±0.65	2.42±0.64	0.430
24 h	2.05±0.66	1.97±0.72	0.309

**Figure 6:**
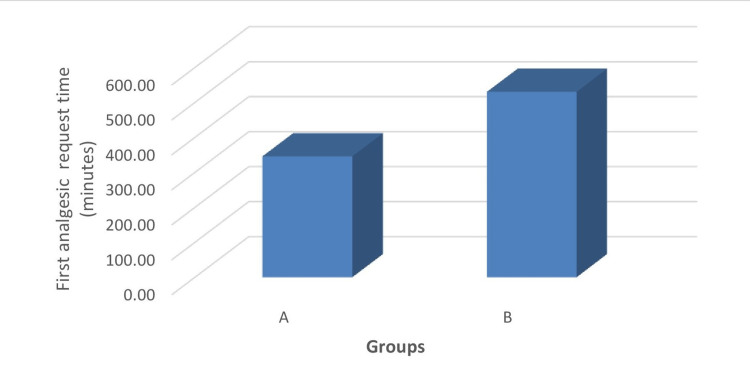
Comparison of time to first analgesic request between the groups.

The duration of analgesia was longer in group B (530.00±326.33 minutes) compared to group A (345.60±252.95 minutes) and the mean values were found to be statistically significant (p<0.05) (Figure 6).

Heart rate, blood pressure, and peripheral oxygen saturation were comparable during the intraoperative and postoperative periods. Nausea was observed in four patients (10.53%) of group A and in two patients (5.26%) of group B and was comparable between the groups (p>0.05) (Table 4). None of the patients had any other side effects like vomiting, bradycardia, hypertension, respiratory depression, pleural puncture, pneumothorax, itching, or any drug-related reactions that happened in the postoperative period.

**Table 4:** Comparison of adverse effects between the groups.

Adverse effect	Group A (n = 38)		Group B (n = 38)		P-value
	Number of patients	%	Number of patients	%	
Nausea	4	10.53%	2	5.26%	0.197
Vomiting	0	0.00%	0	0.00%	-

## Discussion

Effective pain control reduces postoperative stress response, facilitates rehabilitation, and improves recovery from surgery. The benefits of effective regional analgesia techniques include reduced pain intensity, decreased incidence of side effects from systemic analgesics, and improved patient comfort. In the present study, various parameters were assessed like first pass success rate, number of passes, number of attempts, procedure time to perform the block, postoperative pain using the visual analog scale, and duration of analgesia. Patients were also assessed for any complications.

The number of passes was significantly less in ultrasound-guided paravertebral block compared to conventional landmark-based paravertebral block. This was in concordance with the study done by Kallidaikurichi Srinivasan et al. [13], who observed that in the preprocedural ultrasound-guided paramedian technique for spinal anesthesia, the number of passes was less than the conventional landmark-guided midline approach, and the difference in mean values was statistically significant (p = 0.01). Qu et al. [14] reported that the median number of needle passes was less in the USG group (1 (1, 2)) than in the landmark-technique group (3 (2, 4)). The observations were in concordance with our study. Another study performed by Park et al. [15] found that the number of needle passes was significantly lower (1.0 (1.0 to 2.0) vs. (4.5 (2.0 to 7.0)) and the success rate at first pass significantly higher, i.e., 65.0% vs. 17.5% in the ultrasound technique compared with the landmark technique, respectively (P<0.001). Geng et al. [16] reported that in the neuraxial block procedure, the number of passes was significantly less in ultrasound-guided group 3 (0, 5) versus landmark-guided group 5 (3, 10) in elderly patients (P<0.01). Similar observations were also reported in our study. In the present study, the number of attempts was significantly less in ultrasound-guided paravertebral block compared with anatomical landmark-based block. Qu et al. [14] observed that the number of attempts was significantly lower in the ultrasound-guided group (1 (1)) than in the landmark-guided group (2 (1, 2)) in spinal anesthesia (P<0.001). Kallidaikurichi Srinivasan et al. [13] also reported that the routine use of a preprocedural ultrasound-guided paramedian technique for spinal anesthesia had significantly reduced the number of attempts required to achieve entry into the subarachnoid space when compared with the conventional landmark-guided midline approach (P = 0.002). Geng et al. [16] found that the number of insertion attempts in the ultrasound-guided group 1(1, 2) was significantly less when compared with landmark-guided group 2(1, 3) of neuraxial block in elderly patients (P<0.01).

In our study, VAS scores were significantly lower in ultrasound-guided paravertebral block compared to anatomical landmark-based paravertebral block at the second and fourth hours, respectively. Our observations were inconsistent with the study done by Patnaik et al. [11], who observed that the VAS score at the second and fourth hours postoperatively was significantly lower in the USG group as compared with the ALT group using the drug ropivacaine (30 ml, 0.5%) (p<0.05). Another randomized controlled trial study conducted by Aydin [4] observed that the analgesic efficacy of an ultrasound-guided paravertebral block in laparoscopic cholecystectomy was significantly lower in the paravertebral block group receiving 20 ml of 0.5% bupivacaine as compared to the control group (P<0.05). Pei et al. [8] observed the effect of the ultrasound-assisted thoracic paravertebral block using 0.75% ropivacaine (paravertebral block with propofol general anesthesia (PPA) group) to improves analgesia after breast cancer surgery. The VAS scores were significantly lower in the PPA group than in the general anesthesia group, with a mean value of 2 and 3, respectively. Gundost et al. [17] reported that the VAS score in USG-guided thoracic paravertebral block (TPVB) group using 20 ml of 0.5% bupivacaine was significantly lower at rest and on movement compared to the general anesthesia group in laparoscopic cholecystectomy (P<0.05). Borle et al. [18] found that analgesic efficacy of landmark-based paravertebral block using 0.5% bupivacaine (20 mL) during percutaneous nephrolithotomy and observed that the VAS scores on rest (0, 1, 2, and 12 h) and movement (all time points) were lower in the PVB group and were observed to be statistically significant (p<0.05).

In the present study, the time of first analgesic demand was earlier in an anatomical landmark-based paravertebral block than in an ultrasound-guided paravertebral block. Patnaik et al. [11] observed that the time to the first postoperative analgesic request after the block was longer in the USG group (median, 502.5 minutes (range, 195-1440 minutes)) as compared with the ALT group (377.5 minutes (range, 215-1440 minutes)). However, the result was observed as statistically not significant between the groups (p>0.05). The probable reason might be the drug 0.5% ropivacaine with a different volume of 30 ml used in the study. Mohamed et al. [19] observed that the time for first rescue analgesia using 20 ml of 0.25% bupivacaine in landmark-based thoracic paravertebral blocks (PVBs) in patients undergoing modified radical mastectomy was found to be longer (6.48±5.24 h) compared to the control group. Similar observations were also reported in our study. Another study conducted by Tomar et al. [20] observed that the time for first rescue analgesia in landmark-based thoracic paravertebral blocks (PVB) was longer in the group receiving 18 ml of 0.25% plain bupivacaine with a mean value of 402.34±28.12 minutes.

In our study, nausea was observed in four patients (10.53%) of the conventional landmark-based paravertebral group and two patients (5.26%) of the ultrasound-guided paravertebral block group, but the mean values were statistically not significant. None of the participants had any other side effects like vomiting, bradycardia, hypertension, respiratory depression, pleural puncture, pneumothorax, itching, or any drug-related reactions in the postoperative period.

Limitations

The total number of patients in our study was relatively low with a short follow-up period compared to preceding studies. Moreover, we did not perform the bilateral paravertebral block, so the comparison between unilateral and bilateral paravertebral blocks could not be done with respect to their effectiveness and complication rates. Our study involved only patients of elective cholecystectomy due to cholelithiasis; we will not be able to generalize our results to all those having cholecystectomy due to any other reasons. Another limitation is that an experienced anesthetist performed all the USG paravertebral blocks, which may have contributed to a higher success rate for the USG-guided technique. Future studies with a larger number of patients are necessary, including patients with thoracic and abdominal pathologies.

## Conclusions

The present study has shown that the analgesic efficacy of ultrasound-guided paravertebral block is linked with a higher success rate, better postoperative analgesia, and comparatively less complications than the landmark technique in patients undergoing laparoscopic cholecystectomy. We thus recommend that ultrasound-guided PVB is a useful and safer approach than the conventional landmark technique for pain management during elective laparoscopic cholecystectomy. Hence, the USG-guided thoracic PVB technique should be considered a safe and effective alternative treatment to conventional methods for postoperative pain management. Further studies with a greater number of patients to ascertain success rates and complications related to paravertebral techniques can be done in the future.
